# The effects of perceived racism on psychological distress mediated by venting and disengagement coping in Native Hawaiians

**DOI:** 10.1186/s40359-017-0171-6

**Published:** 2017-01-12

**Authors:** Joseph Keawe‘aimoku Kaholokula, Mapuana C.K. Antonio, Claire K. Townsend Ing, Andrea Hermosura, Kimberly E. Hall, Rebecca Knight, Thomas A. Wills

**Affiliations:** 1 Department of Native Hawaiian Health, John A. Burns School of Medicine, University of Hawaii at Manoa, Honolulu, USA; 2 University of Hawaii Cancer Center, Honolulu, USA

**Keywords:** Native Hawaiian, Discrimination, Racism, Coping

## Abstract

**Background:**

Studies have linked perceived racism to psychological distress via certain coping strategies in several different racial and ethnic groups, but few of these studies included indigenous populations. Elucidating modifiable factors for intervention to reduce the adverse effects of racism on psychological well-being is another avenue to addressing health inequities.

**Methods:**

We examined the potential mediating effects of 14 distinct coping strategies on the relationship between perceived racism and psychological distress in a community-based sample of 145 Native Hawaiians using structural equation modeling.

**Results:**

Perceived racism had a significant indirect effect on psychological distress, mediated through venting and behavioral disengagement coping strategies, with control for age, gender, educational level, and marital status.

**Discussion:**

The findings suggest that certain coping strategies may exacerbate the deleterious effects of racism on a person’s psychological well-being.

**Conclusion:**

Our study adds Native Hawaiians to the list of U.S. racial and ethnic minorities whose psychological well-being is adversely affected by racism.

## Background

Psychological distress (i.e., symptoms of depression and anxiety) affects 20 – 30% of adults in developed countries [1, 2]. It is associated with an increased risk for major psychiatric disorders [3–5], high-risk sexual behaviors [6], and cardiovascular disease, stroke, and cancer-related morbidity and mortality [7–10]. In the U.S., the prevalence of psychological distress is higher in indigenous populations — American Indians, Alaska Natives, and Native Hawaiians — compared to other ethnic groups [11–14]. Native Hawaiians, the indigenous people of Hawai‘i, report more depression symptoms than people from other minority ethnic groups [15, 16]. Despite the higher prevalence of psychological distress among indigenous populations compared to other ethnic groups, a dearth of research exists elucidating the factors that contribute to these mental health inequities.

Psychological distress among racial and ethnic minorities, to include indigenous populations, has been partially attributed to their experience of racism [17–19]. Racism is a chronic social stressor defined as the beliefs, acts, and institutional measures that devalue people because of their phenotype or racial and ethnic affiliation [20]. Racism can be manifested in a number of ways, including institutional racism (e.g., in the justice or educational systems) and interpersonal racism (e.g., stigmatization, avoidance, or social exclusion). People subjected to ethnic or racial maltreatment often experience psychological distress due to the unjust, unprovoked, and uncontrollable nature of racism [20]. Although studies have linked racism to psychological distress, a majority of these studies have been conducted with African-Americans [21–25], Hispanics [26], and Asian Americans [27, 28]. Few studies exist among indigenous U.S. populations, despite their indigenous status and a long history of maltreatment (e.g., treaty violations and displacement), compulsory acculturation strategies (e.g., segregation and banning of native languages), and the devaluing of their cultural practices (e.g., banning indigenous cultural and healing practices) [29, 30].

Social stress theory postulates that social sources of stress, such as racism and other types of discrimination, can negatively impact a person’s mental or physical health [31, 32]. Stressors are the external circumstances that challenge the ordinary capacity of an individual or obstruct the individual from obtaining desired ends [33]. Stress is the resulting internal state of arousal that occurs when their capacity to effectively deal with the stressor is taxed beyond one’s available resources [31]. Most vulnerable are individuals from groups assigned to a lower social status, such as many racial and ethnic minority groups and people of lower socio-economic circumstances, who are more likely to be discriminated against and less likely to have the personal resources to effectively deal with such stressors [34]. Meyer et al. [35] examined the social stress hypothesis and found that a disadvantaged social status due to race/ethnicity was associated with higher levels of chronic strain and poorer coping resources. Like most racial and ethnic minorities, Native Hawaiians are at an increased risk of being exposed to racism and are overrepresented in lower socio-economic conditions [30].

The coping strategy a person employs to deal with his or her experience of racism can either serve to buffer against or facilitate its adverse mental health effects [36, 37]. Coping responses to stressors can be divided into two general categories: active versus passive coping strategies [37–42]. A person may use active coping strategies to address his or her stressor by taking actions to modify the situation or seek support from others or his or her religious faith and, thereby, lessen its emotional impact. In contrast, a person may use passive coping strategies by abusing substances, becoming angry, or avoiding the problem. In this case, a passive coping strategy might lead to a racist event being relived (e.g., ruminating) as to prolong the negative emotional response it has on a person. Thus, there could be differential and mutually independent effects between active and passive coping strategies on psychological distress levels in response to racist events. It is for these reasons that coping strategies have been conceptualized as a mediator in the relationship between racism and psychological distress [37, 43], as described in Lazarus and Folkman’s transactional stress model [44] and in Clark et al.’s biopsychosocial model of racism [20]. Several studies using structural equation modeling (SEM) have shown that passive coping strategies, mainly anger expression and avoidance, mediated the relationship between perceived racism and higher levels of psychological distress [45]. Other studies have found that an active coping strategy served to buffer against or lessen the adverse effects of perceive racism on psychological distress [45].

Because racism-related psychological distress is believed to lead to more severe chronic diseases (e.g., hypertension and heart disease) and mental health conditions (e.g., major depression) [25, 46–48], it is imperative to elucidate modifiable factors, such as coping strategies, for intervention. Previous research has already linked perceived racism with hypertension [49], obesity [50], and cortisol dysregulation in Native Hawaiians [51]. Only one study to date has examined the effects of general perceived discrimination (e.g., due to race, ancestry, national origins, skin color, or physical disability) on depressive symptoms in 104 Native Hawaiian adults [52]. They found a significant positive correlation between perceived discrimination and depression. However, no study to date has specifically examined the impact of perceived racism on mental health status, and the role of specific coping strategies, in Native Hawaiians.

Examining the effects of racism on psychological distress and its coping mediators in Native Hawaiians extends this field of inquiry into indigenous populations. In the U.S., a vast majority of empirical research to date in this field has focused on African-Americans, Hispanics, and Asian-Americans. There is a dearth of empirical research on indigenous populations, such as Native Hawaiians, American Indians, and Alaska Natives. Elucidating the mechanism by which racism impacts the mental health of indigenous populations could offer novel insights because they differ considerably in acculturation status, (e.g., native versus immigrant status), historical and political relations with government (e.g., land dispossession and treaty disputes), and notions of assimilation compared to other U.S. ethnic groups [13].

In response, we investigated the relationship between perceived racism and psychological distress in a community-based sample of adult Native Hawaiians. Since previous studies with other ethnic groups demonstrated that specific coping strategies mediate this relationship, we examined the mediating effects of 14 distinct coping strategies (7 active and 7 passive strategies), as measured by the Brief COPE Inventory [53], using structural equation modeling (SEM). We hypothesized that, for Native Hawaiians who generally employ passive rather than active coping strategies, a significant association between perceived racism and psychological distress would be evident, controlling for certain socio-demographic characteristics. No specific hypothesis as to what passive coping strategies would serve as mediators was made. It is important to note that we chose to examine specific coping strategies over aggregating them into the two broad categories of active and passive strategies. The latter approach may fail to detect the effects of a specific coping strategy when aggregated with other less relevant coping strategies.

## Methods

### Study design and participants

We employed a cross-sectional correlational study design to measure perceptions of racism, degree of psychological distress, types of coping strategies commonly used, and socio-demographic characteristics from 145 adult (≥18 years of age) Native Hawaiians recruited from a rural community in Hawai‘i. A Native Hawaiian was defined as any person who is a descendant of the original peoples of Hawai‘i [54]. The majority of the 145 participants were female (71.2%), married (67.8%), and had at least a high school diploma or its equivalent (55.5%). Their mean age was 55.1 (*SD* = 14.0). Table 1 summarizes the participants’ characteristics.

**Table 1:** Participants’ characteristics

Characteristics	Mean (SD) or %
Psychological distress scores	30.8 (14.9)
Perceived racism scores	19.3 (7.7)
Age (years)	54.9 (13.8)
Female (vs. male)	71%
Educational attainment	
No high school diploma	5.5%
High school diploma or equivalent	55.8%
Some college/technical/vocational	27.0%
College graduate	11.7%
Marital Status	
Never married	10.3%
Currently married	67.3%
Divorced/separated/widowed	21.4%
Brief COPE subscale scores	
Active coping	6.0 (1.6)
Emotional support	5.2 (2.0)
Instrumental support	4.9 (2.0)
Religion	6.2 (2.2)
Positive reframing	6.0 (1.7)
Planning	6.0 (1.7)
Humor	3.8 (1.7)
Acceptance	6.5 (1.5)
Venting	4.3 (1.6)
Self-distraction	5.4 (1.7)
Denial	3.6 (2.4)
Behavioral disengagement	3.2 (1.6)
Self-blame	4.1 (1.7)
Substance use	2.4 (1.3)

SD = standard deviation. Due to missing data the sample size for the Brief COPE subscales range from 141 to 145

### Assessment instruments

#### Psychological distress

Psychological distress was measured by aggregating the total scores (after transformation to equivalent scales) from the 10-item Perceived Stress Scale (PSS) [55, 56] and the 10-item Center for Epidemiological Studies — Depression Scale [57] into a single composite measure. The PSS measures perceived stress on a global level over the previous month. Example items include “In the last month, how often have you felt that you were unable to control the important things in your life?” and “In the last month, how often have you felt nervous and ‘stressed’?” with responses ranging from zero (‘never’) to four (‘very often’). The construct validity of the PSS has been demonstrated in different populations with a Cronbach’s alpha of .89 [58, 59]. The CES-D measures cognitive, affective, and behavioral symptoms of depression in which participants rank the frequency of symptoms experienced in the last week. Example items include “I was bothered by things that usually don’t bother me” and “My sleep was restless” with responses ranging from zero (‘rarely or none of the time’) to three (‘to most or all of the time). The use of the CES-D as a valid measure of depressive symptoms among different ethnic groups, including Native Hawaiians, has been supported in several previous studies [53, 60, 61]. The CES-D has been found to have a Cronbach’s alpha of .72 in a previous study of Native Hawaiians [62]. The aggregation of the PSS and CES-D yielded a score range of 0 – 100, with higher scores indicating more psychological distress.

Psychological distress is characterized by symptoms of depression (e.g., sadness and hopelessness), anxiety (e.g., restlessness, nervousness), and other negative emotional responses (e.g., anger and frustration) [63]. The 10-item CES-D captures commonly experienced depression symptoms and the 10-item PSS captures symptoms common to anxiety and anger expression. Since racism is found to impact a person’s mental health in different ways, most often indicated by either symptoms of depression, anxiety, and/or anger and frustration, we wanted to be sure to capture these different forms of psychological distress [64]. The aggregation of these two measures into a composite measure of psychological distress allows for a comprehensive assessment of this higher-order construct. To increase confidence in our composite measure of this higher-order construct, we examined the Pearson’s product moment correlation coefficient for the PSS and CES-D scores in our sample and found it to be .75 (*p* <.0001), suggesting they are highly correlated constructs. We also calculated the Chronbach’s alpha for this aggregate measure based on our sample and found it to be .86, indicating a very good level of internal consistency amongst the PSS and CES-D items.

#### Perceived racism

Perceived racism was measured by a 10-item shortened version of the original 32-item Oppression Questionnaire (OQ) [65]. The 10-item OQ was validated in a previous study of Native Hawaiians to measure perceived racism [51]. Participants were asked how people in power have treated or thought of them and other Native Hawaiians over the past year. The OQ measures two aspects of perceived oppression: 1) *felt* oppression, which considers a person’s subjective experience of feeling oppressed (four items) and 2) *attributed* oppression, which is oppression a person attributes to an oppressive social group (six items). Example items of the *felt* oppressed subscale include “We are not considered to be as good as others” and “My group is often looked down upon.” Example items of the *attributed* oppression subscale include “They keep us from living the way we want” and “Some people look down on me and my group.” Response options ranged from 1 (not at all) to 4 (a great deal). The OQ total score ranges from 10 to 40, with higher scores indicating more perceived racism.

#### Coping strategies

The 28-item Brief COPE [53] was used to measure 14 distinct coping strategies. The Brief COPE was derived from the longer 60-item COPE inventory [40]. It queries a variety of different coping methods (e.g., praying or meditating, receiving emotional support from others, criticizing oneself, etc.) through 14 subscales of two items each. The subscales are 1) active coping, 2) planning, 3) emotional support, 4) instrumental support, 5) religion, 6) positive reframing, 7) acceptance, 8) venting, 9) humor, 10) self-distraction, 11) denial, 12) behavioral disengagement, 13) self-blame, and 14) substance use. Subscales 1 to 7 assess active coping strategies while subscales 8 to 14 assess passive coping strategies. Participants are asked to indicate to what extent they do each item when experiencing a stressful event. Responses are on a 4-point scale and range from 1 (I haven’t been doing this at all) to 4 (I’ve been doing this a lot). The total scores for each subscale range from 2 to 8, with higher scores indicating a greater frequency of using the coping strategy. The Brief COPE has been used extensively in other populations, with Chronbach’s alpha for each subscale ranging from .50 to .90, with nine ≥ .65 [55].

#### Socio-demographic covariates

We obtained socio-demographic data, including sex, age, educational attainment (no high school diploma; high school diploma or its equivalent; some college, technical, or vocational training; or college graduate), marital status (never married; currently married; separated/divorced; or widowed), and self-reported ethnic identification.

### Procedures

Our study was approved by the University of Hawai‘i Institutional Review Board. For more details about the procedures used for this study see Kaholokula et al. [51]. Briefly, the participants for this study were recruited from the database of the Kohala Health Research Project, which was a five-year community-based epidemiological study of diabetes and cardiovascular risk factors [66]. The Kohala Health Research Project’s database had contact information for 494 Native Hawaiian adults (270 females and 224 males). We generated a random list of Native Hawaiian participants for recruitment into our study. From this list, the first 145 participants that could be contacted and agreeable to participation were recruited. A community health nurse assisted with recruitment, which was done by phone and/or mail-out invitations sent to the home addresses on record. The inclusion criteria of the Kohala Health Research Project were as follow: 1) 18 years of age and older, 2) resident of the North Kohala community, and 3), if female, not pregnant. For those who agreed to participate in this follow-up study, informed consent was obtained from each participant and then clinical measures were obtained (e.g., weight and blood pressures) and a battery of questionnaires were administered that included the PSS, CES-D, OQ, and Brief COPE. A $20.00 gift card was given to each participant for their participation.

### Data reduction and statistical analysis

Descriptive statistics were generated using JMP statistical software (version 7.0) with an alpha level of .05. Pearson product-moment correlation coefficients were calculated for all variables. The categorical variables of sex (1 = male; 2 = female), educational attainment (1 = no high school diploma or its equivalent; 2 = high school diploma or its equivalent; 3 = some college, technical, or vocational training; or 4 = college graduate), and marital status (1 never married to 3 = disrupted marital status) were dummy coded for these analyses. To evaluate the internal consistency of the multi-item measures, Cronbach’s alphas were calculated. For the Brief COPE subscales that had a significant bivariate correlation with both perceived racism and psychological distress, scores were entered into a structural equation modeling (SEM) analysis.

SEM was conducted in Mplus [67] to test a mediational model of pathways from perceived racism to psychological distress. The main predictor was perceived racism score, which was specified as exogenous (i.e., not predicted by any prior variable in the model). Potential confounders that could be correlated with perceived racism (respondents’ age, sex, education, and marital status) were also specified as exogenous and their correlations with perceived racism score were all included in the model. Selected scales from the Brief COPE were specified as endogenous (i.e., could be predicted by prior variables in the model) with a residual covariance. The criterion variable was a score for psychological distress.

## Results

### Descriptive statistics

A summary of the descriptive statistics of study variables are shown in Table 1. The mean psychological distress score of 30.8 (*SD* = 14.9) indicates a low to moderate level of distress while the mean perceived racism score of 19.3 (*SD* = 7.7) indicates a moderate to high level of perceived racism in this sample, overall. The Brief COPE subscale scores varied from substance use as the lowest (*M* = 2.4; *SD* = 1.3) to acceptance as the highest (*M* = 6.5; *SD* = 1.5) coping strategy reported.

### Intercorrelation between study variables

Table 2 presents the intercorrelation matrix for all study variables (with the exception of marital status). The perceived racism score had significant positive correlations with positive reframing (*r* = .23, *p* <.01), venting (*r* = .21, *p* <.05), and behavioral disengagement (*r* = .25) scores. Psychological distress score had significant positive correlations with venting (*r* = .32, *p* <.001) and behavioral disengagement (*r* = .37, *p* <.001). Venting and behavioral disengagement scores were significantly correlated with both perceived racism and with psychological distress, thus indicating that they could be possible mediators. The zero-order correlation between the perceived racism score and psychological distress score was non-significant. Age was the only covariate with a significant correlation with psychological distress (*r* = -.21, *p* <0.01). Marital status had a significant association with perceived racism score (*r* = .20, *p* <.02). As discussed by MacKinnon et al. [68], there can be a mediation process even if the exogenous variable does not have a significant zero-order correlation with the criterion.

**Table 2:** Intercorrelation matrix of study variables

Variable	1	2	3	4	5	6	7	8	9	10	11	12	13	14	15	16	17	18	19
1. Perceived racism	─																		
2. Psychological distress	.13	─																	
3. Age	.09	−.24^†^	─																
4. Sex	−.13	.09	−.04	─															
5. Education	−.02	−.07	−.13	−.02	─														
6. Active	.06	−.06	−.06	.02	.03	─													
7. Emotional support	−.01	.07	−.04	.04	.03	.43^±^	─												
8. Instrumental support	−.04	.16	−.05	.04	−.02	.42^±^	.76^±^	─											
9. Religion	.07	−.06	.27^†^	.15	.07	.39^±^	.41^±^	.44^±^	─										
10. Positive reframing	.23^†^	.08	.04	.06	.01	.51^±^	.44^±^	.48^±^	.46^±^	─									
11. Planning	.06	−.08	−13	.10	.01	.65^±^	.46^±^	.55^±^	.42^±^	.55^±^	─								
12. Humor	.12	.16	−.32^±^	−.06	.10	.14	.29^±^	.34^±^	.03	.18*	.27^†^	─							
13. Acceptance	.15	−.09	.17*	.08	−.02	.43^±^	.32^±^	.34^±^	.42^±^	.45^±^	.55^±^	.15	─						
14. Venting	.21*	.32^±^	−.20*	−.05	.02	.20*	.18*	.34^±^	.09	.28^±^	.29^±^	.48^±^	.12	─					
15. Self-distraction	.12	.16	.11	.06	−.06	.33^±^	.23^†^	.25^†^	.40^±^	.40^±^	.31^±^	.24^†^	.27^†^	.36^±^	─				
16. Denial	.02	.27^†^	.03	.11	−.21*	.06	.20*	.23^†^	.07	−.02	.06	.05	.05	.27^†^	.18*	─			
17. Behavioral disengagement	.25^†^	.37^±^	.02	−03	−.24^†^	−.07	.04	.04	−.02	.07	.14	.18*	.17*	.34^±^	.26^†^	.22^†^	─		
18. Self-blame	.14	.33^±^	−.01	−.02	.00	.22^†^	.23^†^	.31^†^	.23^†^	.40^±^	.32^±^	.22^†^	.27^±^	.38^±^	.35^±^	.11	.30^±^	─	
19. Substance use	.06	.20*	−.21	.06	−.03	.14	.16	.13	−.06	.21*	.21*	.30^±^	.08	.42^±^	.17*	.11	.26^†^	.15	─

*p <.05, ^†^ *p* <.01, ^±^ *p* <.001

### Test for indirect effects

**Fig. 1:**
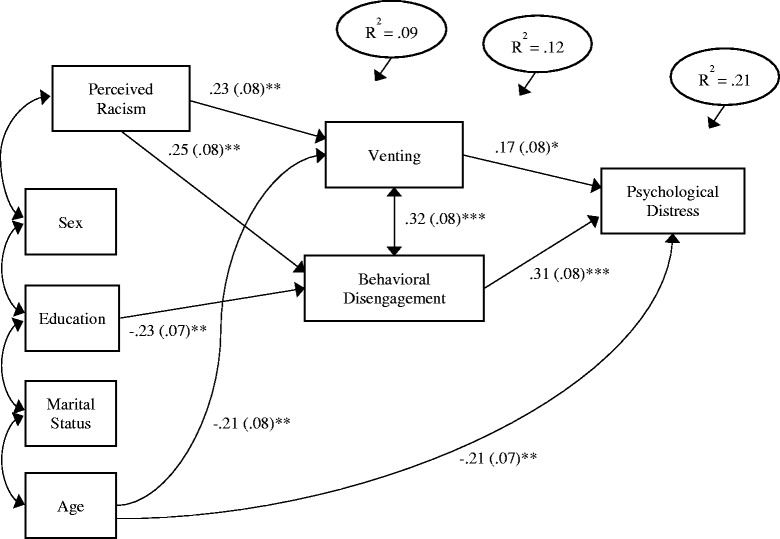
Structural equation model of significant indirect effects for the relationship between perceived racism and psychological distress mediated by venting and behavioral disengagement coping styles with socio-demographic covariates. Standardized coefficient (standard error) is reported for all paths. *p <.05, **p <.01, ***p <.001

A structural model was specified to test for possible indirect effects of racism on psychological distress through coping strategies. Since only venting and behavioral disengagement scores had significant zero-order correlations with the psychological distress score, they were the only endogenous variables entered into the model to test for mediation effects. These variables were specified with a covariance of their residual terms, so any pathways to the criterion variable represent independent effects. All the socio-demographic covariates and their intercorrelations with perceived racism were included as exogenous variables in the model, to control for their effects. The initial model was specified with all paths from the exogenous variables to the mediators, two paths from the mediators to the criterion variable (i.e., psychological distress), and a direct effect from perceived racism to the criterion. The direct path from racism to distress was non-significant and was dropped from the model together with several non-significant paths for demographic variables. After the initial model was estimated, modification indices were examined for direct effects from the socio-demographic variables to the criterion variable and one direct effect was added. In the final model, only significant paths (*p* <.05) were retained. Figure 1 depicts the final model with standardized coefficients and standard errors for all significant paths. Goodness-of-fit tests indicated that this model fit the sample data well [χ^2^ (10, *N =* 145) = 4.55, *p* = .92; Tucker-Lewis Index = 1.14; Comparative Fix Index = 1.0; Root Mean Square Error of Approximation = .000 (90% CI = .000 – .032)]. The coping strategies had a significant residual correlation with each other (*r* = .32, *SE* = .08, *p* <.000). Racism and demographic effects accounted for 9% of the variance in venting and 12% of the variance in behavioral disengagement. Together the variables in the model accounted for 21% of the variance in psychological distress.

Mediation effects were found in the relation between perceived racism and psychological distress, through relations of racism to venting and behavioral disengagement coping strategies. Perceived racism had paths with positive sign to venting (β = .23, *SE* = .08, *p* <.004) and behavioral disengagement (β = .25, *SE* = .08, *p* <.001). In turn, venting (β = .17, *SE* = .08, *p* <.03) and behavioral disengagement (β = .31, *SE* = .08, *p* <.000) had paths with positive sign to psychological distress. The overall indirect effect for racism was significant, Critical Ratio = 3.08, p <.01. In addition to effects observed for the racism variable, education had an inverse path to behavioral disengagement (β = -.23, *SE* = .07, *p* <.002) and age had inverse paths to venting (β = -.21, *SE* = .08, *p* <.006) and to psychological distress (β = -.21, *SE* = .07, *p* <.004).

## Discussion

Our study was the first to examine general coping strategies as mediators in the relationship between perceived racism and psychological distress in a sample of adult Native Hawaiians. The strengths of this study were the use of a non-clinical, community-based sample and the examination of various empirically validated coping strategies. The findings from this study make a contribution to indigenous health by examining the mechanism by which racism, as a chronic social stressor, affects the mental health of an understudied indigenous population. Since chronic psychological distress due to the experience of racism is hypothesized to lead to negative physical health outcomes (e.g., hypertension and heart disease) [69], the amelioration of racism-induced psychological distress in this population can be a viable avenue to reducing the health inequities experienced by other ethnic and racial minorities in the U.S.

Overall, we found that the relationship between perceived racism and psychological distress in our sample of Native Hawaiians occurred largely through indirect effects. Of the 14 coping strategies measured by the Brief COPE [53], we found only venting and behavioral disengagement to mediate the relationship between perceived racism and psychological distress. Higher levels of perceived racism were related to greater use of venting and behavioral disengagement as coping strategies. In turn, higher levels of these two coping strategies were related to higher levels of psychological distress. These associations held across differences in socio-demographic factors, such as age and education level. Thus, our findings support our general hypothesis in which venting and behavioral disengagement, as general passive coping strategies, mediate the relationship between perceived racism and adverse psychological outcomes in Native Hawaiians.

Venting is a form of anger expression and behavioral disengagement is an indicator of learned helplessness (i.e., giving up or withdrawing one’s effort to deal with a stressor) [70, 71]. For Native Hawaiians, the experience of racism is likely to lead to psychological distress for those who have a tendency for anger expression and who “give up” on dealing with the stressors they encounter. Because these coping strategies are considered “passive” coping strategies that do not lead to effective stress management, they are likely to exacerbate and/or carry forward the adverse effects of racism on a person’s psychological wellbeing [71, 72].

Understandably, anger expression is a prevalent coping strategy in dealing with the experience of racism for many racial and ethnic minorities in the U.S. [64]. Anger expression has been found to mediate the relationship between perceived racism and psychological distress in African-Americans [73, 74] and between perceived racism and general health in aboriginal youth of Australia [75]. Brown and colleagues [76] also used the Brief COPE to examine what 14 coping strategies African Americans used in response to racism-specific stressors (situational) versus dispositional coping and also found that venting was commonly used in response to racism. The other racism-specific coping strategy Brown found was religion, which was not the case in our study.

Disengagement as a coping style is less studied than anger coping for dealing with racism. However, Villegas-Gold and Yoo [77] found disengagement coping strategies (i.e., self-criticism, wishful thinking, and social withdrawal) to mediate the relationship between perceived discrimination and subjective well-being in Mexican American college students. Behavioral disengagement, as measured in this study, has more in common with the concept of learned helplessness in which there is a perceived absence of control over a stressor and in effectively dealing with it [78]. Racism has been hypothesized to be a root cause of learned helplessness [79], and a sense of powerlessness in response to racism has been described in African-American women [80, 81].

The findings of our study expand on social stress theory and its emphasis on coping resources by highlighting the mediating role of certain passive coping strategies in the relationship between racism as a social stressor and psychological well-being. Specifically, the notion that “passive” coping strategies, such a sense of helplessness in dealing with the stressor or emotional venting, appears to be the mechanism by which racism may adversely impact an indigenous person’s psychological well-being. It stands to reason that these types of coping strategies only serve to maintain or “relive” the emotional distress (anger or helplessness) elicited by racist acts and offers very little in way of effectively dealing with them. Thus, our finding of a significant indirect path by way of anger expression and disengagement coping, and no direct path from perceived racism to psychological distress, suggest that, for Native Hawaiians, these two types of passive coping strategies are the causal link between racism and psychological distress. This finding also emphasizes the consideration of mediator variables when examining the relationships between perceived racism and health status in Native Hawaiian and other indigenous populations, especially when it appears as though no significant direct relationship exists.

We did not find any mediating role for active coping strategies in our study. As noted, other studies have found that these types of strategies may serve to buffer against or lessen the adverse effects of perceived racism on psychological distress [45]. Again, it could be that the relationship between perceived racism and psychological distress only exists with certain types of passive coping strategies for reasons already mentioned. Another explanation is that our study lacked the statistical power necessary to detect the smaller mediating effects of the active coping strategies measured. Although our findings add to the extant scientific literature in way of identifying specific adverse coping strategies to racist experiences in Native Hawaiians, it does underscore the complex role coping plays and that active and passive coping strategies may not necessarily have opposing effects on psychological distress [37].

In further understanding our finding of no direct relationship between perceived racism and psychological distress in our sample of Native Hawaiians, it could be that the exposure of racism in and of itself may not necessarily lead to psychological distress, but that the distress is activated by harmful coping strategies. It could also be that our sample size was too small to detect a direct effect. However, Antonio and colleagues’ [52] study of Native Hawaiians found a direct effect between general perceived discrimination and depression in a smaller sample of Native Hawaiians. Our study asked only about racial discrimination while the aforementioned study casted a wider net in regards to the types of discrimination measured. It could be that there is a compounding effect of racism and other forms of discrimination on directly influencing a person’s psychological wellbeing and thus linking them directly.

As Alvarez and colleagues [37] indicated, perceived racism is by nature idiosyncratic and multifaceted and, thus, its experience, coping, and impact on psychological wellbeing can differ as a function of the form of racism (e.g., institutional versus interpersonal), the context in which it occurs (e.g., work setting versus public places), and/or its chronicity to name a few. Future research in this area will need to consider such factors to further elucidate the different permutations by which racism impacts psychological wellbeing. In the case of Native Hawaiians and other indigenous populations, our study has provided the foundational support needed to guide future studies in this area.

Several limitations of this study need to be acknowledged. Our sample was mostly female and older adults. It is possible that the results might be different with a more balanced sample in way of gender and age. A study among Filipino Americans did find gender differences in coping mediators in which men tended to use active and support-seeking strategies, while women used avoidance coping [45]. The instructions for the Brief COPE asked the participants to respond, in general, how they deal with stressful events rather than asking specifically about racist events. It is possible that the coping strategies employed for racist events differ from those employed for other types of stressors. Our study may have also lacked statistical power to capture other coping strategies with smaller effect sizes that could have served as mediators. However, it is apparent that we had enough statistical power to capture the mediating effects of venting and behavioral disengagement. Replication with larger samples and other populations would be desirable to test for the generality of the indirect effects observed here.

## Conclusion

There is a need for more research with indigenous populations that investigates the pathways by which racism affects their physical and mental health. Our study adds Native Hawaiians to the list of U.S. racial and ethnic minorities (e.g., African Americans, Hispanics, and Filipinos) who experience a high level of racism and whose health status is adversely affected by it. It also adds to the mounting scientific literature showing the negative health outcomes associated with racism and its contribution to health inequities in the U.S. Ideally, the elimination of racism from society is the desirable outcome. Until this can be achieved, our study points to the need for intervention strategies that focus on developing more active coping strategies in persons’ experiencing a high degree of racism as to lessen its deleterious effects on their psychological well-being.

## References

[1] Bultmann U, Kant I, Kasl S, Beurskens A, Van den Brandt P (2002). Fatigue and psychological distress in the working population psychometrics, prevalence, and correlates. J Psychosom Res.

[2] Kilkkinen A, Kao-Philpot A, O’neil A, Philpot B, Reddy P, Bunker S (2007). Prevalence of psychological distress, anxiety and depression in rural communities in Australia. Aust J Rural Health.

[3] Baum A, Posluszny D (1999). Health psychology: mapping biobehavioral contributions to health and illness. Annu Rev Psychol.

[4] Cohen S, Janicki-Deverts D, Miller G (2007). Psychological stress and disease. JAMA.

[5] McEwen B (2007). (2007). Physiology and neurobiology of stress and adaptation: central role of the brain. Physiol Rev.

[6] Diclemente R, Wingood G, Crosby R, Sionean C, Brown L, Rothbaum B (2001). A prospective study of psychological distress and sexual risk behavior among black adolescent females. Pediatrics.

[7] Carney RM, Freedland KE (2002). Psychological distress as a risk factor for stroke-related mortality [editorial]. Stroke.

[8] Lewis T, Everson-Rose S, Powell LH, Matthews KA, Brown C, Karavolos K (2006). Chronic exposure to everyday discrimination and coronary artery calcification in African-American women: the SWAN heart study. Psychosom Med.

[9] Russ T, Stamatakis E, Hamer M, Starr J, Kivimaki M, Batty G (2012). Association between psychological distress and mortality: individual participant pooled analysis of 10 prospective cohort studies. BMJ.

[10] Salomon K, Jagusztyn N (2008). Resting cardiovascular levels and reactivity to interpersonal incivility among black, Latina/o, and white individuals: the moderating role of ethnic discrimination. Health Psychol.

[11] Nutrition, Physical Activity and Obesity Data, Trends and Maps web site. U.S. Department of Health and Human Services, Centers for Disease Control and Prevention (CDC), National Center for Chronic Disease Prevention and Health Promotion, Division of Nutrition, Physical Activity and Obesity, Atlanta, GA, 2015. Available at http://www.cdc.gov/nccdphp/DNPAO/index.html.

[12] Harris K, Edlund M, Larson S (2005). Racial and ethnic differences in the mental health problems and use of mental health care. Med Care.

[13] Kaholokula JK, Nacapoy AH, Dang K (2009). Social justice as a public health imperative for kanaka maoli. Alternative.

[14] Kim G, Bryant A, Parmelee P (2012). Racial/ethnic differences in serious psychological distress among older adults in California. Int J Geriatr Psychiatry.

[15] State of Hawaii, Department of Health. Hawaii Behavioral Risk Factor Surveillance System. 2013. http://health.hawaii.gov/brfss/.

[16] State of Hawaii, Department of Health. Hawaii Behavioral Risk Factor Surveillance System. 2007. http://health.hawaii.gov/brfss/.

[17] Castor ML, Smyser MS, Taualii MM, Park AN, Lawson SA, Forquera RA (2006). A nationwide population-based study identifying health disparities between American Indians/Alaska natives and the general populations living in select urban counties. Am J Public Health.

[18] Stephens C, Porter J, Nettleton C, Willis R (2006). Disappearing, displaced, and undervalued: a call to action for indigenous health worldwide. Lancet.

[19] Williams DR, Mohammed SA (2009). Discrimination and racial disparities in health: evidence and needed research. J Behav Med.

[20] Clark R, Anderson NB, Clark VR, Williams DR (1999). Racism as a stressor for African Americans. Am Psychol.

[21] Pieterse AL, Todd NR, Neville HA, Carter RT (2012). Perceived racism and mental health among black American adults: a meta-analytic review. J Couns Psychol.

[22] Smith V, Stewart T, Myers A, Latu I (2008). Implicit coping responses to racism predict African Americans’ level of psychological distress. Basic Appl Soc Psych.

[23] Thomas AJ, Witherspoon KM, Speight SL (2008). Gendered racism, psychological distress, and coping styles of African American women. Cultur Divers Ethnic Minor Psychol.

[24] Williams DR, Yu Y, Jackson J (1997). Racial difference in physical and mental health: social-economic status, stress and discrimination. J Health Psychol.

[25] Williams DR (1999). Race, socioeconomic status, and health the added effects of racism and discrimination. Ann N Y Acad Sci.

[26] Todorova IL, Falcón LM, Lincoln AK, Price LL (2010). Perceived discrimination, psychological distress and health. Sociol Health Illn.

[27] Alvarez A (2002). Racial identity and Asian Americans: support and challenges. New Dir Stud Serv.

[28] Gee GC, Spencer MS, Chen J, Takeuchi D (2007). A nationwide study of discrimination and chronic health conditions among Asian Americans. Am J Public Health.

[29] Kaholokula JK, Culbertson P, Agee MN, Makasiale C (2007). Colonialism, acculturation and depression among kanaka maoli of Hawai‘i. Penina uliuli: confronting challenges in mental health for pacific peoples.

[30] Kaholokula JK, Nacapoy AH, Dang KL (2009). Social justice as a public health imperative for Kānaka Maoli. Alternative: Int J Indigenous Peoples.

[31] Aneshensel CS (1992). Social stress: theory and research. Annu Rev Sociol.

[32] Pearlin L (1989). The sociological study of stress. J Health Soc Behav.

[33] Pearlin L, Kaplan HB (1983). Role strains and personal stress. Psychosocial stress: trends in theory and research.

[34] Bolger N, Zuckerman A (1995). A framework for studying personality in the stress process. J Pers Soc Psychol.

[35] Meyer I, Schwartz S, Frost D (2008). Social patterning of stress and coping: Does disadvantaged social statuses confer more stress and fewer coping resources?. Soc Sci Med.

[36] Brondolo E, Brady ver Halen N, Pencille M, Beatty D, Contrada R (2009). Coping with racism: a selective review of the literature and a theoretical and methodological critique. J Behav Med.

[37] Alvarez AN, Liang CTH, Molennaar C, Nguyen D, Alvarez AN, Liang CTH, Neville HA (2016). Moderators and mediators of the experience of racism. The cost of racism for people of color: contextualizing experiences of discrimination.

[38] Billings AG, Moos RH (1981). The role of coping responses and social resources in attenuating the stress of life events. J Behav Med.

[39] Billings AG, Moos RH (1984). Coping, stress and social resources among adults with unipolar depression. J Pers Soc Psychol.

[40] Carver C, Scheier M, Weintraub J (1989). Assessing coping strategies: a theoretically based approach. J Pers Soc Psychol.

[41] Moos RH, Billings AG, Goldberger L, Breznitz S (1982). Conceptualizing and measuring coping resources and processes. Handbook of stress: theoretical and clinical aspects.

[42] Snow-Turek A, Norris M, Tan G (1996). Active and passive coping strategies in chronic pain patients. Pain.

[43] Liang C, Alvarez A, Juang L, Liang M (2007). The role of coping in the relationship between perceived racism and racism-related stress for Asian Americans: gender differences. Am J Psychol.

[44] Lazarus RS, Folkman S (1984). Stress, appraised, and coping.

[45] Alvarez AN, Juang LP (2010). Filipino Americans and racism: a multiple mediation model of coping. J Couns Psychol.

[46] Cassidy C, O’connor RC, Howe C, Warden D (2004). Perceived discrimination and psychological distress: the role of personal and ethnic self-esteem. J Couns Psychol.

[47] Paradies Y (2006). A systematic review of empirical research on self-reported racism and health. Int J Epidemiol.

[48] Pascoe EA, Richman LS (2009). Perceived discrimination and health: a meta-analytic review. Psychol Bull.

[49] Kaholokula JK, Iwane MK, Nacapoy AH (2010). Effects of perceived racism and acculturation on hypertension in native Hawaiians. Hawaii Med J.

[50] McCubbin L, Antonio M (2012). Discrimination and obesity among native Hawaiians. Hawaii Med J.

[51] Kaholokula JK, Grandinetti A, Keller S, Nacapoy AH, Kingi TK, Mau MK (2012). Association between perceived racism and physiological stress indices in native Hawaiians. J Behav Med.

[52] Antonio MCK, Hyeong JA, Townsend Ing C, Dillard A, Cassel K, Kekauoha BP, Kaholokula JK. The effects of perceived discrimination on depression in Native Hawaiians. Hawaii J Med Public Health. In press.

[53] Carver C (1997). You want to measure coping but your protocol’s too long: consider the brief cope. Int J Behav Med.

[54] Office of Hawaiian Affairs (2006). Native Hawaiian databook 2006.

[55] Cohen S, Kamarck T, Mermelstein R (1983). (1983). A global measure of perceived stress. J Health Soc Behav.

[56] Cohen S, Williamson G, Spacapan S, Oskamp S (1988). Perceived stress in a probability sample of the United States. The social psychology of health: Claremont symposium on applied social psychology.

[57] Radloff L (1977). The CES-D scale: a self-report depression scale for research in the general population. Appl Psychol Meas.

[58] Cole S (1999). Assessment of differential item functioning in the perceived stress scale-10. J Epidemiol Community Health.

[59] Roberti J, Harrington L, Storch E (2006). Further psychometric support for the 10-item version of the perceived stress scale. J Coll Couns.

[60] Beekman ATF, Deeg DJH, Van Limbeek J, Braam AW, de Vries MZ, van Tilburg W (1997). Criterion validity of the center for epidemiologic studies depression scale (CES-D): results from a community based sample of older adults in the Netherlands. Psychol Med.

[61] Hertzog C, Alistine JV, Usala PD, Hultsch DF, Dixon R (1990). Measurement properties of the center for epidemiological studies depression scale (CES-D) in older populations. Psychol Assess.

[62] Kaholokula JK, Braun KL, Kana‘iaupuni S, Grandinetti A, Chang HK (2006). Ethnic-by-gender differences in cigarette smoking among Asian and pacific islanders. Nicotine Tob Res.

[63] Mirowsky J, Ross CE (2002). Selecting outcomes for the sociology of mental health: issues of measurement and dimensionality. J Health Soc Behav.

[64] Kaholokula JK, Alvarez AN, Liang CTH, Neville HA (2006). Physical health correlates. Contextualizing the cost of racism for people of color: theory, research and practice.

[65] Victoroff J. The Oppression Questionnaire [online]. 2005. http://www.humiliationstudies.org/documents/VictoroffOppressionQuestionnaire.pdf.

[66] Grandinetti A, Kaholokula JK, Theriault AG, Mor JM, Chang HK, Waslien C (2007). Prevalence of diabetes and glucose intolerance in an ethnically diverse rural community of Hawaii. Ethn Dis.

[67] Muthen LK, Muthen BO (2005). Mplus User’s guide.

[68] MacKinnon DP, Fairchild AJ, Fritz MS (2007). Mediation analysis. Annu Rev Psychol.

[69] Garnefski N, Teerds J, Kraaij V, Legerstee J, Van Den Kommer T (2004). Cognitive emotion regulation strategies and depressive symptoms, differences between males and females. Pers Individ Dif.

[70] Martin R, Dahlen E (2005). Cognitive emotion regulation in the prediction of depression, anxiety, stress, and anger. Pers Individ Dif.

[71] Lazarus RS (1993). Coping theory and research: past, present, and future. Psychosom Med.

[72] Brondolo E, Thompson S, Brady N, Appel R, Cassells A, Tobi JN (2005). The relationship of racism to appraisals and coping in a community sample. Ethn Dis.

[73] Nyborg V, Curry J (2003). The impact of perceived racism: psychological symptoms among African American boys. J Clin Child Adolesc Psychol.

[74] Pittman CT (2011). Getting mad but ending up sad: the mental health consequences for African Americans using anger to cope with racism. J Black Stud.

[75] Priest N, Paradies Y, Stewart P, Luke J (2011). Racism and health among urban aboriginal young people. BMC Public Health.

[76] Brown T, Phillips C, Abdullah T, Vinson E, Robertson J (2011). Dispositional versus situational coping: Are the coping strategies African Americans use different for general versus racism-related stressors?. J Black Psychol.

[77] Villegas-Gold R, Yoo H (2014). Coping with discrimination among Mexican American college students. J Couns Psychol.

[78] Alloy L, Peterson C, Abramson L, Seligman M (1984). Attributional style and the generality of learned helplessness. J Pers Soc Psychol.

[79] Fernando S (1984). Racism as a cause of depression. Int J Soc Psychiatry.

[80] Vines A, Baird D, McNeilly M, Picciotto I, Light K, Stevens J (2006). Social correlates of the chronic stress of perceived racism among black women. Ethn Dis.

[81] Thomas SA, Gonzalez-Prendes AA (2009). Powerlessness, anger, and stress in African American women: implications for physical and emotional health. Health Care Women Int.

